# Multicellular 3D models to study myocardial ischemia–reperfusion injury

**DOI:** 10.3389/fcell.2024.1494911

**Published:** 2024-11-15

**Authors:** Merel Peletier, Xiaohan Zhang, Scarlett Klein, Jeffrey Kroon

**Affiliations:** ^1^ Department of Experimental Vascular Medicine, Amsterdam Cardiovascular Sciences, Amsterdam UMC Location University of Amsterdam, Amsterdam, Netherlands; ^2^ Amsterdam Cardiovascular Sciences, Atherosclerosis and Ischemic Syndromes, Amsterdam, Netherlands; ^3^ Laboratory of Angiogenesis and Vascular Metabolism, VIB-KU Leuven Center for Cancer Biology, Leuven, Belgium; ^4^ Laboratory of Angiogenesis and Vascular Metabolism, Department of Oncology, KU Leuven and Leuven Cancer Institute (LKI), Leuven, Belgium

**Keywords:** ischemia–reperfusion, 3D models, organoids, cardiac tissue, endothelial cell, cardiomyocyte

## Abstract

Coronary heart disease is a major global health threat, with acute myocardial ischemia–reperfusion injury (IRI) being a major contributor to myocardial damage following an ischemic event. IRI occurs when blood flow to ischemic tissues is restored and exacerbates the cellular damage caused by ischemia/hypoxia. Although animal studies investigating IRI have provided valuable insights, their translation into clinical outcomes has been limited, and translation into medical practice remains cumbersome. Recent advancements in engineered three-dimensional human *in vitro* models could offer a promising avenue to bridge the “therapeutic valley of death” from bench to bedside, enhancing the understanding of IRI pathology. This review summarizes the current state-of-the-art cardiovascular 3D models, including spheroids, organoids, engineered cardiac microtissues, and organ-on-a-chip systems. We provide an overview of their advantages and limitations in the context of IRI, with a particular emphasis on the crucial roles of cell–cell communication and the multi-omics approaches to enhance our understanding of the pathophysiological processes involved in IRI and its treatment. Finally, we discuss currently available multicellular human 3D models of IRI.

## 1 Introduction

Myocardial ischemia–reperfusion injury (IRI) refers to the cellular damage or dysfunction of cardiac tissue that occurs when blood flow and oxygen supply are restored in the myocardium, following acute coronary syndrome (ACS), a period of restricted or blocked cardiac blood flow. IRI is a complex condition that can manifest in various forms, including microvascular obstruction, lethal myocardial reperfusion injury, reperfusion-induced arrhythmias, and myocardial stunning (Fröhlich et al., 2013). This reoxygenation-induced damage can contribute up to 50% of the final infarct size and can lead to chronic heart failure and death (Yellon and Hausenloy, 2007).

The pathological mechanisms underlying IRI are multifaceted. In general, during ischemia, oxygen-deprived cardiac tissue shifts toward anaerobic metabolism, particularly in cardiomyocytes, which normally obtain their energy primarily through oxygen-dependent fatty acid oxidation (FAO). This metabolic shift leads to increased lactic acid production and results in intracellular metabolic acidosis, ion imbalance, and cell swelling. When oxygen levels return upon reperfusion, the rapid influx of calcium into the cardiomyocytes leads to an excessive production of reactive oxygen species (ROS) that can result in local cell damage and microvascular obstruction (Buja and Vander Heide, 2016; Heusch, 2020). Although ROS sensitizes the mitochondrial permeability transition pore (MPTP), the substantial increase in calcium levels in the mitochondrial matrix is responsible for MPTP opening. The opening of the MPTP occurs during the first minutes of reperfusion and is a crucial factor in IRI. It results in the collapse of the mitochondrial membrane potential and uncoupling of oxidative phosphorylation with subsequent ATP depletion, which significantly contribute to cardiomyocyte death and infarct size (Buja and Vander Heide, 2016; Heusch, 2020; Ong et al., 2015).

Despite the significant progress in understanding IRI, many aspects remain unclear and continue to be the focus of ongoing research (Fröhlich et al., 2013; Hausenloy and Yellon, 2013). For example, calcium overload is a crucial factor at the onset of IRI, but how calcium fluxes between different cellular compartments, like the sarcoplasmic reticulum, mitochondria, and cytosol, is not fully mapped yet. Additionally, while the opening of the MPTP plays a key role in aggravating reperfusion injury, the precise role of various triggers remains to be discovered. Although multiple forms of cell death, including apoptosis, necroptosis, and ferroptosis, are all involved in IRI, necrosis appears to be the most prominent, especially closest to the ischemic core of the infarct (De Villiers and Riley, 2020). However, the relative importance and contributions of each form of cell death remain largely unclear. Furthermore, it is currently unknown which form of cell death predominates at different stages of injury or in different cell types within the heart. Finally, the environmental factors and genetic predispositions that render individuals more susceptible to IRI than others require further investigation (Hausenloy and Yellon, 2013; Zhang et al., 2024).

Despite these still unfulfilled gaps in the knowledge of IRI, significant progress has been made in the medical treatment of ACS, including the polypill, used as the secondary prevention tool after the first event (Castellano et al., 2022). However, mortality rates within the first year after an ACS event remain alarmingly high and vary between 6% (Steen et al., 2022) and 10% (Ulvenstam et al., 2023). Moreover, the prevalence of adverse outcomes and long-term health implications, including infarct size and progressive cardiac dysfunction, which can ultimately result in chronic heart failure, remains substantial. Notably, there are currently no clinically approved, effective treatments available that alleviate short-term complications like myocardial stunning, arrhythmias, no-reflow phenomenon, and IRI-induced cell death (Hausenloy and Yellon, 2013). The low success rate of clinical trials has not only contributed to the already high cost of drug discovery, which could lead to a reduced interest from pharmaceutical companies in pursuing research and development in this area, but it has also been proposed that improved preclinical screening methods could potentially identify around 70% of cardiac toxicities observed in clinical trials (Olson et al., 2000). Preclinical studies in animals have played an important role in advancing our understanding of disease mechanisms and in the development and testing of therapeutic compounds. In rodents, the commonly used technique of ligation of the left arterial descending coronary artery is used to test potential cardioprotective strategies prior to their application in larger animal models (De Villiers and Riley, 2020). Before moving to human clinical trials, studies are conducted in canine, ovine, porcine, and non-human primate models due to their increased physiological similarity and predictive value, as reviewed in detail elsewhere (Rahman et al., 2023). However, despite the success of certain compounds in preclinical trials, such as MTP-131 to target oxidative stress (Gibson et al., 2016), cyclosporin A to target MPTP opening (Ottani et al., 2016), or carperitide to promote vasodilatation (Suwa et al., 2005), they were all tested in rodent and canine models but showed low translatability in clinical trials. Part of this is attributable to genetic, molecular, and cellular variations in the cardiovascular system between humans and animals (De Villiers and Riley, 2020; Milani-Nejad and Janssen, 2014). To overcome part of these limitations of non-human models while mimicking the complexity of multiple cell types in one system, advanced cardiac 3D models, using human induced pluripotent stem cell cardiomyocytes (hiPSC-CMs) to study IRI, are currently being developed at a fast pace.

In conclusion, while IRI is currently incurable and its pathology causes considerable damage to the heart after cardiac arrest, its multifactorial nature and multiple unanswered questions require further development of models investigating the disease. Because of its rapid advancement, this review focuses on human multicellular 3D culture systems in the study of IRI.

## 2 Local interactions in IRI

**FIGURE 1:**
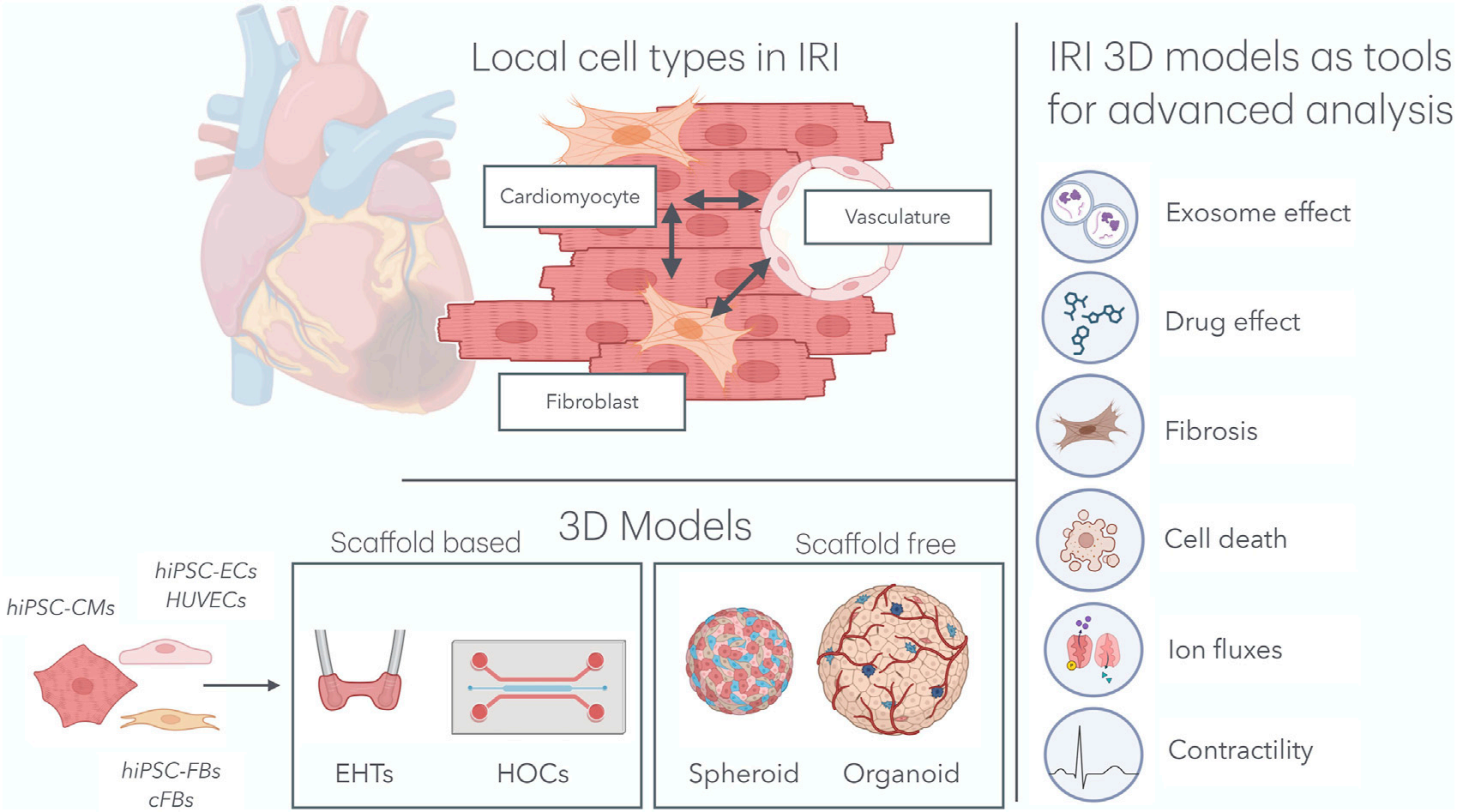
Multicellular organ-on-a-chip technology for IRI research. **Top left:** In the cardiac tissue, hiPSC-CMs, hiPSC-FBs, and the cardiac microvasculature interacts directly with one another, mimicking native tissue architecture. **Bottom left:** Various combinations of cell types, including hiPSC-CMs, hiPSC-ECs, HUVECs, hiPSC-FBs, and cFBs, have been used to model IRI in both scaffold-based 3D systems (e.g., EHTs and HOCs) and scaffold-free systems (e.g., spheroids and organoids). **Right:** These models have been subjected to IRI environments, providing key insights into the effects of exosomes and drugs on the progression of IRI, as well as deepening our understanding of cardiomyocyte cell death, fibrosis, ion fluxes, and contractility. IRI, ischemia–reperfusion injury; hiPSC-CMs, human induced pluripotent stem cell-derived cardiomyocytes; hiPSC-FBs, human induced pluripotent stem cell-derived fibroblasts; hiPSC-ECs, human induced pluripotent stem cell-derived endothelial cells; HUVECs, human umbilical vein endothelial cells; cFBs, cardiac fibroblasts; EHT, engineered heart tissue; HOCs, heart-on-chips.

Although cardiomyocytes constitute the majority of the cardiac volume, they account for only approximately 50% of the total number of cells (Litviňuková et al., 2020). Given the complex and multifaceted nature of IRI, it is essential to consider the role of other local cell types that significantly influence the aggravation and resolution of IRI, particularly endothelial cells (ECs) and cardiac fibroblasts (cFBs) (Figure 1).

### 2.1 Cardiac fibroblasts

cFBs are geometrically interspersed between cardiomyocytes and, in normal circumstances, lead to extracellular matrix (ECM) homeostasis in the cardiac tissue. Immediately after IRI, activated fibroblasts differentiate into myofibroblasts, triggering an initially protective form of cardiac fibrosis aimed at preserving structural integrity. Fibrotic EMC remodeling and inflammatory activation can lead to long-term cardiac complications due to excessive scarring (Hinz et al., 2012). Myofibroblast differentiation is driven by the expression of contractile genes, such as *ACTA2*, which encode for smooth-muscle α-actin (α-SMA) (Tallquist and Molkentin, 2017). Myofibroblasts can worsen local inflammation by activating the NLRP3 inflammasome response (Sandanger et al., 2013). Once differentiated, myofibroblasts increase the ECM, deposition factors like collagen -I, -III, -IV, -V, and -VI, glycoproteins such as fibronectin and tenascin-C, and proteoglycans, all responsible for the healing and scarring processes, as reviewed in detail elsewhere (Tallquist and Molkentin, 2017; Kanisicak et al., 2016). The scarring process is necessary for maintaining the structural integrity and functionality of the heart. However, excessive fibrosis can result in adverse remodeling, increased stiffness, reduced contractility, and, ultimately, heart failure (Zhang et al., 2024).

### 2.2 Cardiac vasculature

ECs are more than just a protective barrier controlling the exchange of nutrients and gasses to the cardiac muscle. In a stress and inflammatory setting, ECs regulate the adhesion and transmigration of immune cells, contributing to the inflammatory response within the cardiac tissue. During IRI, ECs play an important role in the aggravation of and protection against IRI. ECs impair vasodilation by reducing NO production, increasing vascular permeability inflammation that could contribute to microvascular obstruction, one of the hallmarks of IRI. Additionally, in the context of IRI, ECs may drive tissue remodeling and hypertrophy after ischemia, as reviewed by Yang et al. (2015) and Singhal et al. (2010). EC-derived extracellular vesicles (EC-EVs) play a crucial role in the alleviation of IRI (Yadid et al., 2020; Li et al., 2023), illustrated by a ∼50% increase in cardiomyocyte viability after an IRI stimulus when EC-EVs were added (Yadid et al., 2020). Mechanistically, Liu et al. discovered that exosomes, a small subtype of extracellular vesicles, derived from human-induced pluripotent stem cell-derived endothelial cells (hiPSC-ECs) restored the expression and activity of reticulum Ca^2+^ ATPase 2a (SERCA-2a) and ryanodine receptor (RYR2) in cardiomyocytes. Consequently, intracellular Ca^2+^ transient and cardiomyocyte contractions were enhanced after MI (Li et al., 2023).

When developing an appropriate research model to study the pathophysiology of IRI, it is crucial to consider both beneficial and deleterious local interactions. Incorporating these interactions will enable the creation of an *in vitro* model that more accurately reflects the human *in vivo* environment.

## 3 Usage of hiPSC-CMs in IRI research

Due to the challenges in obtaining cells from the adult human heart—stemming from limited accessibility, poor proliferation capacity, and reduced viability in culture—alternative approaches are often required. Development of hiPSC-CMs has emerged as a valuable alternative, although their immature nature currently limits translation into clinical practice. Although hiPSC-CMs exhibit inherent genetic and epigenetic variations across different lines to a greater extent than non-iPSC cultures, this allows for easier genetic disease models and opportunities for personalized medicine (Zhan et al., 2023; Stein et al., 2021).

The use of immature hiPSC-CMs in IRI modeling, using a relatively prolonged period of hypoxia, 8–24 h and subsequent reoxygenation of 24 h, has been observed to elicit alterations in several key parameters, including the beating rate, polarization time, field potential, and sarcomere structure (Häkli et al., 2021). Recent work using a 2D culture of hiPSC-CMs revealed the upregulated expression of early growth response 1 (ERG1) in IRI-induced apoptosis, an effect that could be suppressed using miR-124-3p (Yang et al., 2024), revealing the possibility of using 2D hiPSC-CMs for simple IRI modeling.

However, critical for IRI, hiPSC-CMs rely primarily on glucose metabolism, making them more resistant to hypoxia and reperfusion than adult cardiomyocytes, which mainly utilize fatty acid oxidation and can lead to reduced accuracy in modeling IRI (Wu et al., 2021; Vučković et al., 2022). As reviewed by Vučković et al. (2022), hiPSC-CMs can be subjected to different maturation protocols in order to partially overcome these important metabolic differences, reporting up to 2–4-fold increase in fatty acid oxidation and a subsequent 50% reduction in glycolysis, more similar to the metabolic phenotype of primary cardiomyocytes. Metabolic maturation accomplished by the supplementation of the media with fatty acids while removing glucose as a substrate leads to up to ∼30% cell death post-hypoxia and reoxygenation, compared to only ∼5% in immature iPSC-CMs, highlighting the translational importance of cell maturation (Hidalgo et al., 2018). Although hiPSC-CMs currently represent the only widespread alternative for modeling human cardiomyocytes *in vitro*, metabolic maturation from glucose to oxidative phosphorylation dependency is an important feature for increasing the accuracy of the induced IRI (Wu et al., 2021; Feyen et al., 2020). These more matured hiPSC-CMs are, among others, characterized by increased sarcomere length (approximately 1.6 μm–2.2 μm), increased upstroke velocity (15–50 V/s to 230–400 V/s), the presence of T-tubules, and increased quantity of mitochondria, which are responsible for their metabolic shift (Wu et al., 2021). Although the use of mature hiPSC-CM 2D models allow for the investigation of basic cell behavior, they do not fully recapitulate the *in vivo* dynamic microenvironment structures and different cell types of the heart, hence limiting the ability to accurately reflect the complex pathophysiology of IRI (Ahmed et al., 2020). In the next section, we categorize the current advances in both scaffold-based and scaffold-free 3D culture systems containing hiPSC-CMs.

## 4 Advances in scaffold-based models

### 4.1 Engineered heart tissues

**Engineered heart tissues (EHTs)** are 3D structures typically created by mixing hiPSC-CMs with a hydrogel into a casting mold, providing a highly reproducible tissue structure. CMs in EHTs align along the force lines of the cell or culture they are placed on, such as rings (Katare et al., 2010), sheets—as used by Yadid et al. (2020), measuring 3.2 mm × 4.2 mm—or elastomeric pillars (Ahmed et al., 2020; Arslan et al., 2022; Mastikhina et al., 2020). Mastikhina et al. (2020) included fibroblasts in their cardiac EHT and investigated the fibrotic effects after ischemia using 2.5*10^5 cells placed between two polydimethylsiloxane (PDMS) pillars. The CMs created a coherent beating syncytium that allows for the detailed measurement of contractile function, including force generation and electrophysiological properties. EHTs are generally easy to produce and can be manufactured on a relatively large scale; however, due to the lack of flow, they are prone to the development of necrotic inner tissue due to oxygen deprivation (Stein et al., 2021).

### 4.2 Heart-on-chips

**Heart-on-a-chip** models are based on a microfluidic system that enables dynamic culture, perfusion, and the addition of a vascular channel (Arslan et al., 2022; Paz-Artigas et al., 2023). Heart-on-chips utilize a range of biocompatible natural and synthetic materials, including hydrogels, (3D-printed) synthetic polymers such as PDMS, or extracellular matrix, to provide structural support and guide cell alignment and organization. These materials allow for the precise spatial arrangement of cell culture chambers, fluidic channels, and measuring devices, such as electrodes (Liu et al., 2020; Paloschi et al., 2021). Nevertheless, heart-on-a-chip systems require specialized equipment, like microfluidic pumps, and can be equipped with biosensors, like microelectrode arrays or intracellular electrodes, for measuring extracellular field potentials and action potentials (Liu et al., 2020) or mechanical biosensors, like force transducers or cantilevers, to measure contractile forces (Yadid et al., 2020). The chip to be cultured on, the microfluidic pump system, and potential sensors add technical complexity and costs compared to 2D and scaffold-free 3D systems.

### 4.3 Spheroids

**Spheroids** belong to the scaffold-free models, together with organoids. Spheroids are simple micro-size 3D aggregates often generated by hanging drop or ultra-low-attachment plate methods (Paz-Artigas et al., 2023; Cho et al., 2021). They are generated from hiPSCs alone or in a mixture with ECs, FBs, or even smooth muscle cells (SMCs). These cells can exhibit synchronized contractions, which may be perceived as beating heart tissue. Regarding their compact nature, they are at an increased risk of O_2_-mediated cell death; this feature can be used to recreate differences in oxygenation, which can be translated to gradients of IRI damage (Richards et al., 2020).

### 4.4 Organoids

**Organoids** are more complex, self-organizing 3D structures that mimic the structural and functional properties of the heart without the need for external support to maintain mechanical integrity (Kaushik et al., 2018). Organoids are made using cells that grow in a 3D structure that resembles the organ in structure and function; this development is similar to the mesodermal development of the human heart (Song et al., 2024; Hofbauer et al., 2021a). These effects can be enhanced by the addition of BMP, VEGF, FGF, and TGF-beta-containing medium, creating organoids consisting of further differentiated cells into different lineages. Adding these factors led to further development of other cell types of the heart, reflected by a significant increase in endothelial VE-cadherin expression, increasing from 4% to 15%. Additionally, CD90 positivity, a fibroblast marker, increased from 6% to 27% when compared to CaO of a similar age and size (Song et al., 2024). Organoids are more complex structures than spheroids and more closely resemble the human heart (Gunti et al., 2021). However, they are more challenging to generate and may exhibit greater structural and functional heterogeneity.

## 5 3D systems to investigate ischemia–reperfusion injury

As discussed previously, not only cardiomyocytes but the whole cardiac tissue including the vasculature and cardiac fibroblasts play an important role in the aggravation and resolution of IRI. Only 3D systems containing multiple cell types are discussed further. The initiation of IRI can be achieved by changing local oxygen concentrations (Yadid et al., 2020; Hidalgo et al., 2018) using a chemical ischemic stimulus of time or by establishing a physical oxygen gradient (Richards et al., 2020). Additionally, cardiac cryoinjury can be used to investigate healing and ECM accumulation in an IRI-like setting in organoids consisting of mostly hiPSC-CMs (Voges et al., 2017) or organoids containing hiPSC-CMs, cFBs, and ECs (Hofbauer et al., 2021a). Both multicellular scaffold-free (Mastikhina et al., 2020; Richards et al., 2020; Song et al., 2024; Richards et al., 2017) and scaffold-based (Yadid et al., 2020; Veldhuizen et al., 2022; Veldhuizen et al., 2020) setups are currently being developed to investigate IRI. All models containing multiple human cell types in an IRI setting are summarized in Table 1.

**TABLE 1:** Current multicellular 3 d models to investigate ischemia–reperfusion injury.

Reference	Model type	Cell types used	Ischemic/reperfusion stimulus	Ischemia-reperfusion readout	Main finding/model characteristics
Song et al. (2024)	Organoid	hiPSC-CMs, hiPSC-ECs, and hiPSC-FBs	Ischemia: 50 uM cobalt chloride, no glucose, high calcium ion 75 h	Cell death (TUNEL 2-fold increase, caspase-3 2-fold increase)	IRI in organoids leads to electrophysiological abnormalities, cardiac fibrosis, and disrupted calcium ion handling
Yadid et al. (2020)	EHT	iPSC-CMs and HUVECs (extracted exosomes)	Ischemia: 1% O_2_, 3 h. High-acidity, low-glucose medium reperfusion: 3 h	2-fold cell death (EthD-1/Hoechst)	EEVs taken up by CMs are protective against Human IRI—EEVs reduced cell death, partial conservation of the proteome, and normalized contractile IRI stress
Veldhuizen et al. (2022)	Heart-on-chip	hiPSC-CM cFBs	Ischemia: 1% O_2_, 24 h Reperfusion: 1 h and 24 h	Increased beating variability, 2-fold lactate increase, and cell death	Fibrosis, cell toxicity, sustained contractile irregularities, sustained lactate levels, and gene expression
Richards et al. (2020)	Organoid	iPSC-CMs, hCFs, HUVECs, and hADSCs	Ischemia: 10% O_2_, 10 days + adrenergic stimulation via norepinephrine	TUNEL staining and decreased NADH autofluorescence	Organoid development similar to human ischemia on the transcriptomic level including pathological calcium handling
Ellis et al. (2022)	Heart-on-chip	hiPSC-CMs hiPSC-ECs	Ischemia: 0.1% O_2_, 3 h Reperfusion: 3 h	miRNA (miR-208b and miR-499) increase similar to IRI *in vivo*	Revealed the potential of miRNA biomarkers for IRI diagnosis, similar to *in vivo*. Changed exosome surface markers
Sebastião et al. (2020)	Spheroid	iPSC-CMs, HUVECs (conditioned media)	Ischemia: < 0.4% O_2_, no glucose, high Na^+^ lactate, low pH (6.8) 5 h. Reperfusion: 16 h	Sarcomere filament disruption and apoptosis at core	Increase in the inflammatory, migrational, and angiogenic proteome. Conditioned media on HUVECs lead to increased angiogenesis

### 5.1 Scaffold-based models

Although some previous IRI-on-chip models were developed utilizing hiPSC-CMs (Paz-Artigas et al., 2023), Yadid et al. (2020) advanced the field by creating a multicellular model consisting of an EHT assembled on a flexible chip mimicking ventricular-like muscle. Although indirect, this model was designed to investigate IRI on a microfluidic platform (Yadid et al., 2020). A flexible cantilever chip was used to study the effects of exosomes on hiPSC-CM survival and function. The CM cantilever chip consists of micro-patterned films to facilitate hiPSC-CM alignment, creating an organized CM sheet more similar to a naïve myocardium than to a 2D monolayer. Hypoxia was induced by exposing the cells to a 1% O₂ environment for 3 h, combined with glucose-depleted and slightly acidified media (pH 6.4) to simulate the ischemic event. This was followed by 1.5 h under normal culture conditions to mimic the reperfusion phase. Exosome production was approximately 6.5 times higher in hypoxic EC-EXs than in normoxic EC-EXs. Additionally, the heart cantilever receiving EC-EXs 3 h prior to ischemia demonstrated improved cell survival and enhanced twitch stress recovery compared to non-EX-treated controls (Yadid et al., 2020). Although the uptake was equal, the positive results could not be replicated when neonatal rat cardiomyocytes were stimulated using human EC-EXs. These results could indicate a species-specific effect of human EC-EXs.

The effect of EVs in IRI was exploited further by Ellis et al. (2022). Their heart-on-a-chip model comprising hiPSC-CMs and hiPSC-ECs was used to investigate the potential role of extracellular vesicle-derived miRNAs in the context of IRI. During ischemia, about 3.5 times more EC-EXs were secreted than during reperfusion or non-ischemic controls; these results were validated in clinical plasma samples. During reperfusion, an anion exchange membrane (AEM)-based miRNA sensor was used to investigate the presence of miRNA in chip effluents compared to clinical controls. miRNAs miR-208 and miR-499 were identified in the model effluent, and they were also present in clinical IRI samples, implicating the potential of this IRI-on-a-chip model for biomarker discovery (Ellis et al., 2022).

In mice, the administration of EC-EVs intranasally every day the first 3 days and twice a week up to 3 weeks after IRI has been demonstrated to improve cardiac function in mice after a 28-day period (Wang et al., 2023). The impact of EVs on reperfusion injury depends on their source. EVs obtained not only from ECs but also isolated from the ischemic heart were administered via an intracardiac injection into a second heart just before reperfusion exacerbated IRI in mice. Further analysis revealed increased M1 polarization of macrophages, as well as increased local cytokine expression (Ge et al., 2021). The development of technologies such as IRI-on-a-chip (Yadid et al., 2020; Ellis et al., 2022) and EHT fibrosis models (Mastikhina et al., 2020) could potentially enable a more detailed investigation of the content, secretion, and effects of these EVs in human models. These advancements offer an opportunity to more comprehensively explore potential therapeutic targets or biomarkers associated with EVs.

Veldhuizen et al. (2020) developed a microdevice with a heart-on-a-chip configuration and surface topographical patterning to facilitate two-dimensional cell alignment. This microfluidic chip was constructed from PDMS and filled with hiPSC-CMs and cFBs at a ratio of 4:1 in an ECM comprising a collagen/fibronectin (Veldhuizen et al., 2020) or collagen/matrigel (Veldhuizen et al., 2022) mixture. These cells formed aligned tissues around the embedded microposts. Culturing on the PDMS chip led to the increased maturation of hiPSC-CMs compared to 2D control, as evidenced by the upregulation of genes involved in calcium uptake and release, including *HCN1*, *KCNQ1*, *CAV1*.*2*, *CAV3*.*1*, *PLN*, and *RYR2* (Veldhuizen et al., 2020). Placing this model in a hypoxic setting (1% O₂ for 24 h), followed by reperfusion for either 1 or 24 h, revealed no change in cell death during hypoxia. However, a significant increase in cell death was observed following oxygen reperfusion at both the 1-h and 24-h time points, indicating the successful induction of reperfusion-related damage (Veldhuizen et al., 2022). Furthermore, 24 h of hypoxia led to a near 2-fold increase in lactate secretion, which is comparable to human physiological levels during IRI, where ischemia only caused minor fluctuations in the inter-beat interval, while reperfusion led to a notable increase in inter-beat variability. Additionally, reperfusion also led to increased expression of α-SMA (Veldhuizen et al., 2022). This *de novo* expression of α-SMA is a hallmark of myofibroblast activation, allowing for the formation of stress fibers and the production of extracellular matrix, which induces fibrosis and scarring (Hinz et al., 2012). The transcriptomic analysis revealed a notable increase in glycolysis and other metabolic pathways, accompanied by a reduction in oxidative phosphorylation (OXPHOS). Collectively, similar pathways and matching functional readouts compared to human ischemia serve as validation for IRI-on-a-chip models and allow for further developments of these 3D-IRI-on-a-chip models (Veldhuizen et al., 2022).

### 5.2 Scaffold-free models

Although both scaffold-based and scaffold-free models allow for the co-culture of cardiomyocytes with different cell types like EVs and cFBs, an acute limitation of scaffold-free models, both organoids and spheroids, is the lack of a functional vascular network to facilitate nutrient exchange and waste removal (Homan et al., 2019), especially in modeling the reperfusion phase, where fast nutrient/oxygen exchange and waste removal are warranted. In static circumstances, only spheroids with a diameter of less than 150 μm have been used to study cardiac ischemia (Richards et al., 2017) as in this size, passive oxygen diffusion is still possible. Small organoids or spheroids are usually less complex and exhibit greater variability in their properties and reduced contractile function than their larger (600 μm) counterparts (Hoang et al., 2021).

Richards et al. (2020) used the absence of a vascular network as a means of gradually introducing ischemia. In their study, 300-μm cardiac organoids were subjected to a 10-day, 10% oxygen + adrenergic stimulation via norepinephrine. The quantification of oxygen diffusion in these microtissues revealed a reduction to 1% oxygen at the core of the organoid. Not only did the low-oxygen treatment result in significant cell death, visualized by TUNEL staining, in accordance with metabolic responses in IRI, but a reduction in non-mitochondrial respiration and an increase in glycolysis during ischemia were also observed. A meta-analysis of transcriptomic changes in these human cardiac infarct organoids revealed significant similarities with the transcriptomes of acute post-infarct tissues from animal models and human cardiac samples affected by ischemic cardiomyopathy. Gene Ontology terms between control and infarcted organoids revealed changes in pathways indicative of altered calcium handling, such as ion transport, calcium signaling, and arrhythmogenic right ventricular cardiomyopathy. A reduction in well-studied calcium handling components, including *ATP2A2, RYR2, CACNA1C*, and *SLC8A1*, was observed, and the peak calcium ion concentration, crucial for CM contraction, was reduced in the interior of the infarcted organoid compared to the edge or control CMs. In addition to altered calcium handling, bulk RNA sequencing analysis revealed changes in pathways related to fibrosis, with genetic alterations mirroring those observed in the infarcted mouse heart. Functionally, there was a notable increase in myofibroblast-like structures (α-SMA + fibroblasts) and a significant increase in tissue stiffness, following infarction (Richards et al., 2020). To conclude, this model provides a valuable opportunity to investigate the mechanisms of fibrosis and calcium fluxes, and it may also serve as a model for exploring druggable targets in ischemia. However, a significant limitation of this model is that due to the distance to the center of the microtissue, even under normoxic conditions, oxygen levels do not exceed 6%, limiting effective reperfusion and potentially affecting the study of reperfusion injury.

As an indirect co-culture containing both iPSC-CMs and human umbilical vein endothelial cells (HUVECs), Sebastião et al. (2020) developed cardiac spheroids, measuring approximately 260 μm, which were cultured for 18 days. The cardiac hiPSC-CM spheroids were subjected to a 5-h-long ischemic protocol, comprising both nutrient and oxygen deprivation with low pH and increased lactic acid supplementation, resulting in sarcomere disturbances and a reduction in viability in the spheroid core. The angiogenic potential of HUVECs was enhanced when stimulated with conditioned media from IRI spheroids, as opposed to hiPSC-CM control media. The present study reveals the indirect, angiogenic effect of the IRI spheroid secretome on the vasculature.

In intestinal organoids, IRI has successfully been modeled to recapitulate properties of *in vivo* IRI responses while reaching a larger size between 200 μm and 400 μm (Kip et al., 2021). One of the defining characteristics of intestinal organoids is their formation of a lumen-enclosed structure, which allows for more effective oxygen differentiation. Although not yet deployed in IRI, the development of cardioids, a specific type of organoid that self-organizes into chamber-like structures (Hofbauer et al., 2021b), could offer the potential to work with scaffold-free models while maintaining proper perfusion.

Recent advantages in the synergic integration of the flow dynamics of the heart-on-a-chip model, together with the self-organizing capacity of cardiac organoids, revealed great advancements over static models and could facilitate the use of larger, more stable and more complex organoids. In a kidney organoid model with fluidic culturing, transcriptomic analysis revealed 229 signaling pathways not identified in the static model (Hiratsuka et al., 2024). Min et al. (2024) revealed the impact of flow EHTs comprising hiPSC-CMs, ECs, and CFs within a 3D heart extracellular matrix hydrogel using a microfluidic chip. Placing these large 1-mm EHTs in a chip system, providing them with nutrient flow, led to increased oxygen concentrations within the cardiac tissues and reduced expression of cleaved caspase-3 in the organoid, compared to no-flow or flowing the organoids in a plate. Functionally, the application of flow on a microfluidic chip resulted in an increase in sarcomere length, the contraction amplitude, both indicators of hiPSC-CM maturation, and a heart rate of 76.59 ± 15.7 beats per minute (BPM) within the frequency range of a healthy human heart. RNA sequencing revealed significant differences between static and plate-flowed EHTs, with an observed increase in genes related to heart development, extracellular matrix organization, and angiogenesis. The relative mRNA expression of CM (*MYH7* and *TNNT2*), EC (*VWF*), and cFB markers (*COL1A1* and *PDGFRA*) increased in the chip-flow model compared to the no- or plate flow models, revealing increased functional differentiation of different cell types (Min et al., 2024). Altogether, the combination of EHTs with chip-based flow enables the functional maturation of diverse cell types within organoids while simultaneously facilitating an increase in size up to 1 mm.

In conclusion, a meta-analysis of transcriptomic changes in human ischemic organoids revealed a strong resemblance to post-infarct murine hearts while still preserving key human-specific characteristics (Richards et al., 2020). The utility of organoids in IRI research is currently constrained by the absence of a vascular network, which is a crucial element in reperfusion studies. However, recent developments in organoid technology have led to the creation of organoids containing chamber-like structures, which have opened new avenues of research in the field of organoid-ischemia–reperfusion studies (Hofbauer et al., 2021b). Moreover, the integration of chip flow, intersecting scaffold-free self-organization and complexity with scaffold-based nutrient exchange (Min et al., 2024), presents promising opportunities for bridging the knowledge and treatment gap in IRI.

## 6 Limitations and future opportunities

### 6.1 Maturation of hiPSC-CMs

As the knowledge on maturation is rapidly evolving, mature hiPSC-CMs will come closer to but remain different from adult human CMs (Ahmed et al., 2020; Tu et al., 2018). Relative immaturity of hiPSC-CMs can lead to reduced translatability of IRI findings compared to more established animal models, potentially limiting their predictive accuracy for human outcomes.

Microtissues comprising hiPSC-CMs and cFBs demonstrated enhanced electrophysiology and contractility superior sarcomere structure and augmented mitochondrial respiration, compared to hiPSC-CMs alone (Giacomelli et al., 2020). In addition, the amplitude of intracellular calcium flux during the contraction–relaxation cycle of hiPSC-CMs increased when co-cultured with cFBs (King et al., 2022), indicating enhanced cardiomyocyte maturation. Both fibroblasts and the co-culture of hiPSC-CMs with ECs result in the increased maturation of hiPSC-CMs (Giacomelli et al., 2020; Abecasis et al., 2019; Gisone et al., 2022; Liu et al., 2021), resulting in more organized and longer sarcomeres than the single-culture controls (Gisone et al., 2022), a feature suggesting a more adult-like phenotype and often associated with improved contractile function (Skorska et al., 2022). Additionally, the co-culture resulted in the increased expression of proteins involved in the deposition of several extracellular matrix components such as collagens and fibronectin (Abecasis et al., 2019), increased the contractility and higher expression of the ventricular cardiomyocyte marker *IRX4* (Liu et al., 2021), and reduced the beating frequency (King et al., 2022).

Both the integration of multiple cell types and functional heart-on-a-chip integration of electrical pacing capabilities (Schneider et al., 2022) or mechanical stretch/stress (Mozneb et al., 2024) can significantly increase hiPSC-CM maturation. A recently developed multi-cell heart-on-a-chip model using multi-cell-type hiPSC-CMs and HUVECs could incorporate both dynamic fluid flow (shear stress) and biomechanical cyclic stretch. The combination of maturation strategies led to an improvement in the functional and transcriptional maturity of hiPSC-CMs, an improvement in the alignment of hiPSC-ECs grown on a heart chip and the facilitation of the formation of a tube-like EC network (Mozneb et al., 2024). Although hiPSC-CMs have yet to reach the maturity or mitochondrial capacity of adult CMs (Wu et al., 2021), it becomes increasingly feasible to combine multiple maturation methods to optimize hiPSC-CMs for modeling IRI in this fast progressing field.

### 6.2 Addition of 3D immune response

One limitation of current 3D IRI models is the absence of the immune component as an injury-response mediator. Recently, Ze Lu et al. not only developed a heart-on-a-chip system that incorporates HUVECs, CFs, and hiPSC-CMs but also tested the addition of an immune fraction in the form of human peripheral blood mononuclear cells (PBMCs) within the vascular channel that initiates migration through the system under the appropriate conditions. This high-throughput setup allows for the straightforward collection of flow data and the measurement of cardiac redouts, such as beating force (Ze Lu et al., 2024). The introduction of SARS-CoV-2 to the 3D system did not result in significant alterations in the secretion of the cytokines IL-6, IL-8, and MCP-1, beating force, or contraction slope, while the addition of PBMCs caused significant alterations in these readouts. Although it has not been tested in an IRI setting, the incorporation of an immune component could potentially enhance physiological relevance of the 3D culturing system.

### 6.3 Optimizing IRI protocols for 3D cardiac models

It is important to recognize the differences in *in vitro* protocols modeling IRI as the methods for inducing both ischemia and reperfusion can vary significantly between studies. This variation highlights the complexity of accurately modeling IRI *in vitro* and underscores the need for careful consideration when comparing findings across different experimental approaches. With oxygen modulation varying between 0.1% (Ellis et al., 2022) and 10% (Richards et al., 2020) and the time subjected to ischemia or reperfusion varying between 1 and 24 h, even at similar oxygen concentrations (Yadid et al., 2020; Veldhuizen et al., 2022), the majority of studies seek to achieve a total cell death rate between 20% and 50%.

Although oxygen deprivation is frequently used to induce ischemia, other physiological variables, like the lack of nutrients and the accumulation of cellular waste products, can also be introduced into the models to induce IRI. For instance, the accumulation of lactic acid during ischemic conditions (20 mM sodium lactate) and the corresponding reduction in pH 6.4 to 6.8 (Sebastião et al., 2020), instead of the physiological range of 7.2–7.4, combined with nutrient and oxygen deprivation, can be utilized to induce IRI in spheroids (Sebastião et al., 2020) and EHTs (Yadid et al., 2020; Chen and Vunjak-Novakovic, 2019). Hidalgo et al. (2018) compared oxygen reduction only to oxygen reduction together with physiological changes in pH and glucose availability during the ischemic episode in mature H9-NCX1+ CMs. A reduction in pH to 6.2, in conjunction with a 2-h period of 0% oxygen and 0 mM glucose, resulted in an approximate 60% increase in the death of CMs *in vitro*, measured by LDH concentration, compared to CMs only treated with 0% oxygen (Hidalgo et al., 2018).

To date, caution is still warranted when comparing and interpreting different IRI results as protocols are optimized per model as the field develops. Rapid developments in the field of hiPSC-CM maturation, as well as further development and standardization of IRI models, are expected to improve the consistency and reliability of IRI modeling *in vitro*.

### 6.4 Technological advancements

Increased sensitivity and opportunities in functional electrophysiological readouts offer great opportunities in IRI development. Integrated live oxygen sensor integration (Schneider et al., 2022) and the integration of patterned intra- and extracellular electrodes on a heart-on-a-chip model (Liu et al., 2020) allow for the measurement of extracellular beating frequency, spatial waveform propagation, and precise action potential measurement, even in an IRI environment (Liu et al., 2020).

Progression of readout techniques has facilitated further progress in this field; Gao et al. pioneered using a multi-omics approach in an IRI setting (Gao et al., 2023). By integrating bulk and single-nucleus RNA sequencing with metabolomics profiling of reperfused rat hearts at various time points post-MI, it was discovered that early reperfusion reduced myocardial IRI by preserving fatty acid metabolism, a process regulated by PPARα. Functionally, pretreatment with the PPARα agonist fenofibrate upregulated genes associated with fatty acid oxidation and TCA pathways and revealed significantly upregulated PPARα expression, indicating that fenofibrate maintains energy metabolism post-infarct. These results were confirmed in animal experiments showing smaller infarct size and reduced fibrosis compared to non-fenofibrate-treated controls (Gao et al., 2023). Although yet to be used in organoid models, spatial transcriptomics combined with single-cell RNA-seq in embryonic hearts has enabled the generation of a three-dimensional cellular map, used to investigate cardiac organoid physiology and behavior in depth (Asp et al., 2019). Omics technology is becoming increasingly extensive and more readily available (Babu and Snyder, 2023) as cellular interactions and specific metabolic changes can be mapped in more detail, paving the way for future scientific advancements and therapeutic strategies.

## 7 Conclusion

Although these new models are a major leap forward that can help increase our insights into IRI pathology, it remains a challenge to fully recapitulate the complexity of human IRI *in vitro*. Consequently, we remain reliant on animal IRI studies to complement the findings obtained in 3D systems. Nevertheless, in light of the rapid advancement of bioprinting, tissue engineering, and microfluidics, along with the growing use of multi-omics testing, these 3D models offer invaluable tools for drug, biomarker, and discovery research, surpassing conventional 2D systems while maintaining human relevance. As the physiological relevance of the 3D models is enhanced by increasing physiological cues and different cell types, data extraction is facilitated by the use of multi-omics and more complex electrophysiological readouts. Furthermore, 3D models offer distinctive advantages in terms of human relevance, availability, experimental control, and reproducibility, making them a valuable addition to animal studies in addressing the significant unanswered questions and facilitating a drug-testing platform in IRI.

## Abbreviations

3D, three-dimensional; ACS, acute coronary syndrome; BPM, beats per minute; CaO, cardiac organoid; cFB, cardiac fibroblast; EC, endothelial cell(s); EC-EVs, endothelial cell-derived extracellular vesicles; ECM, extracellular matrix; EEV, endothelial extracellular vesicle; EHT, engineered heart tissue; EV, extracellular vesicle; FAO, fatty acid oxidation; FBs, fibroblasts; HOC, heart-on-a-chip; hiPSC-CM, human-induced pluripotent stem cell-derived cardiomyocytes; hiPSC-EC, human-induced pluripotent stem cell-derived endothelial cells; hiPSC-FB, human-induced pluripotent stem cell-derived fibroblasts; HUVEC, human umbilical vein endothelial cell; IRI, ischemia–reperfusion injury; MPTP, mitochondrial permeability transition pore; MYH7, myosin heavy chain 7; OXPHOS, oxidative phosphorylation; PDMS, polydimethylsiloxane; PBMC, peripheral blood mononuclear cell; PPARα, peroxisome proliferator-activated receptor alpha; ROS, reactive oxygen species; RYR2, ryanodine receptor 2 (gene); SMCs, smooth muscle cells; TUNEL, terminal deoxynucleotidyl transferase dUTP Nick end labeling; α-SMA, alpha smooth muscle actin.
